# Applied anatomy of female pelvic plexus for nerve-sparing radical hysterectomy(NSRH)

**DOI:** 10.1186/s12905-023-02651-2

**Published:** 2023-10-10

**Authors:** Fan Ye, Hongyu Su, Hang Xiong, Wenxin Luo, ZiHeng Huang, Guoqing Chen, Hongying Zhou

**Affiliations:** 1 Department of Human Anatomy, West China School of Basic Medical Sciences and Forensic Medicine, Sichuan University, Chengdu, Sichuan China; 2 West China School of Stomatology, Sichuan University, Chengdu, China; 3 Sichuan Provincial People’s Hospital, School of Medicine, University of Electronic Science and Technology of China, Chengdu, Sichuan China

**Keywords:** Pelvic plexus, Hypogastric nerve, Vesical venous plexus, Nerve-sparing radical hysterectomy (NSRH)

## Abstract

**Background:**

Nerve-sparing radical hysterectomy(NSRH)has the advantage of reducing postoperative complications and improving postoperative quality of life. The separation and protection of the pelvic plexus in NSRH is extremely important and challenging.

**Methods:**

24 female cadaveric hemipelves were dissected. Morphologic patterns and compositions of pelvic plexus as well as relationship of pelvic plexus to the surrounding structures were observed and documented.

**Results:**

Two patterns of superior hypogastric plexus were observed, including fenestrated and cord-like shape. The origin of bilateral hypogastric nerves were inferiorly to upper margin of promontory about 1.6 ± 0.1 cm and parallel to the ureter in front of the sacrum. Pelvic splanchnic nerves(PSN)from the second sacral nerve, the third sacral nerve and the forth sacral nerve were observed combing with the hypogastric nerves within the lateral rectal ligament. The sacral sympathetic trunk can be identified anteriorly or medially to the anterior sacral foramen. We identified the boundaries of pelvic plexus as following: the upper margin is formed by the PSNs from the third sacral nerve, posterior margin by inferior rectal artery, and anteriorly by vesical venous plexus. The uterine branches from pelvic plexus were observed accompanying with uterine artery, while other branches were inferiorly to the artery. The PSNs were located beneath the deep uterine veins within the cardinal ligament. The upper margin of pelvic plexus was observed directly approach to urinary bladder within the vesico-vaginal ligament as a single trunk accompanying with ureter, between the middle and inferior vesical veins.

**Conclusions:**

Our study clarified the intricate arrangement, distribution and relationship of female pelvic plexus and the related structures to provide reference index for NSRH application. The innervation patterns of bladder and uterine were clarified, and by tracing these visceral branches of pelvic plexus, we suggest several new important land markers for NSRH.

## Background

The pelvic autonomic nervous system comprises the superior hypogastric plexus (SHP) and the inferior hypogastric plexus (IHP) [1]. The SHP, located below the aorta bifurcation, descends into the pelvis and receives the L3 and L4 splanchnic nerves. It then divides into left and right hypogastric nerves upon entering the pelvis [2]. The IHP receives the hypogastric nerves (HN) and is composed of the pelvic visceral nerves (PSN) and the sacral sympathetic trunks (SST). Situated in the retroperitoneal space next to the pelvic viscera, the hypogastric plexus is a combination of sympathetic and parasympathetic fibers [3]. The pelvic autonomic nervous system pertains to the innervation and control of the pelvic organs, including the bladder, reproductive organs, and rectum, injury to this nervous system during pelvic surgery can lead to a range of dysfunctions.

Nerve-sparing Radical hysterectomy (NSRH) has attracted attention for its ability to minimize complications and improve prognosis, However, the small and intricate structure of autonomic nerve branches in the pelvis poses a certain challenge to surgeries. NSRH is typically used for in cervical cancer patients with FIGO stage IA2, IB1, and IIA1 cervical cancer, without lymph node metastasis or invasion of surrounding tissue [4, 5]. During the surgical treatment, may damage the pelvic plexus and its branches will be damaged more or less, resulting in a series of anatomical complications, including bladder dysfunction, sexual dysfunction and rectal dysfunction [6]. Proper nerve protection during radical hysterectomy in patients with benign lesions such as endometriosis improves dyschezia, dyspareunia, chronic pelvic pain and gastrointestinal function, but there is still a risk of urinary dysfunction [7, 8]. The most common long-term complication of NSRH is bladder dysfunction due to nerve systems innervating the lower urinary tract may be disrupted [9, 10]. Postoperative complications caused by pelvic nerve injury seriously interfere with the quality of life of patients [11, 12]. In this study, we accumulated detailed anatomical data of the pelvic plexus and its branches through gross dissection of the female pelvic structures to provide reference indicators for the application of NSRH.

## Materials and methods

### Specimen collection

12 formalin-fixed adult female corpses with no history of pelvic surgery, normal development, and intact pelvic tissues and organs were chosen. The specimens were obtained from the Department of Anatomy, West China School of Basic Medicine and Forensics, Sichuan University. This study was approved by the ethics committee of the Sichuan University (Chengdu, China).

### Dissection protocol

#### Pretreatment of specimens

The female cadavers were cross-sectioned at the level of the third lumbar vertebra, and the thighs were truncated at the upper 1/4 on both sides. The anterior abdominal wall was cut to fully expose the abdominal and pelvic structures, the structures below the aortic bifurcation were kept intact.

#### Anatomical methods of pelvic nerves

After opening the pelvic peritoneum, the abdominal aorta and the reticulated abdominal aortic plexus on both sides and in front of it were found. Then, continue to trace down, a fenestrated or cord-like nerve bundle can be seen, which is the SHP, the plexus continues to descend into two symmetrical nerves before the sacrum below the sacral promontory, namely the left and right hypogastric nerve.

The HNs were kept tracing down along the lateral pelvic wall to expose the IHP. The connective tissues were removed and the communicating branches of the second sacral nerve, the third sacral nerve and the fourth sacral nerve (S2, S3, S4) involved in the hypogastric plexus were separated. The sacral sympathetic trunk was dissected using the same method. The pelvic plexus was carefully lifted, the branches of the pelvic plexus that innervate the bladder, uterus, and rectum were separated respectively, the nerves were cleared as much as possible.

During this process, the morphology of the superior hypogastric plexus and its relationship to the aortic bifurcation and the abdominal midline were studied. The origin and course of the right and left hypogastric nerves were recorded, and the distances from their origin to the upper edge of the promontory were measured. The course and contribution to the pelvic plexus of the sacral splanchnic and pelvic splanchnic nerves were documented. The composition and branching of pelvic plexus as well as the relationship of pelvic plexus to the surrounding structures were documented.

## Results

### Superior hypogastric nerve (SHP) and hypogastric nerve (HN)

**Fig. 1:**
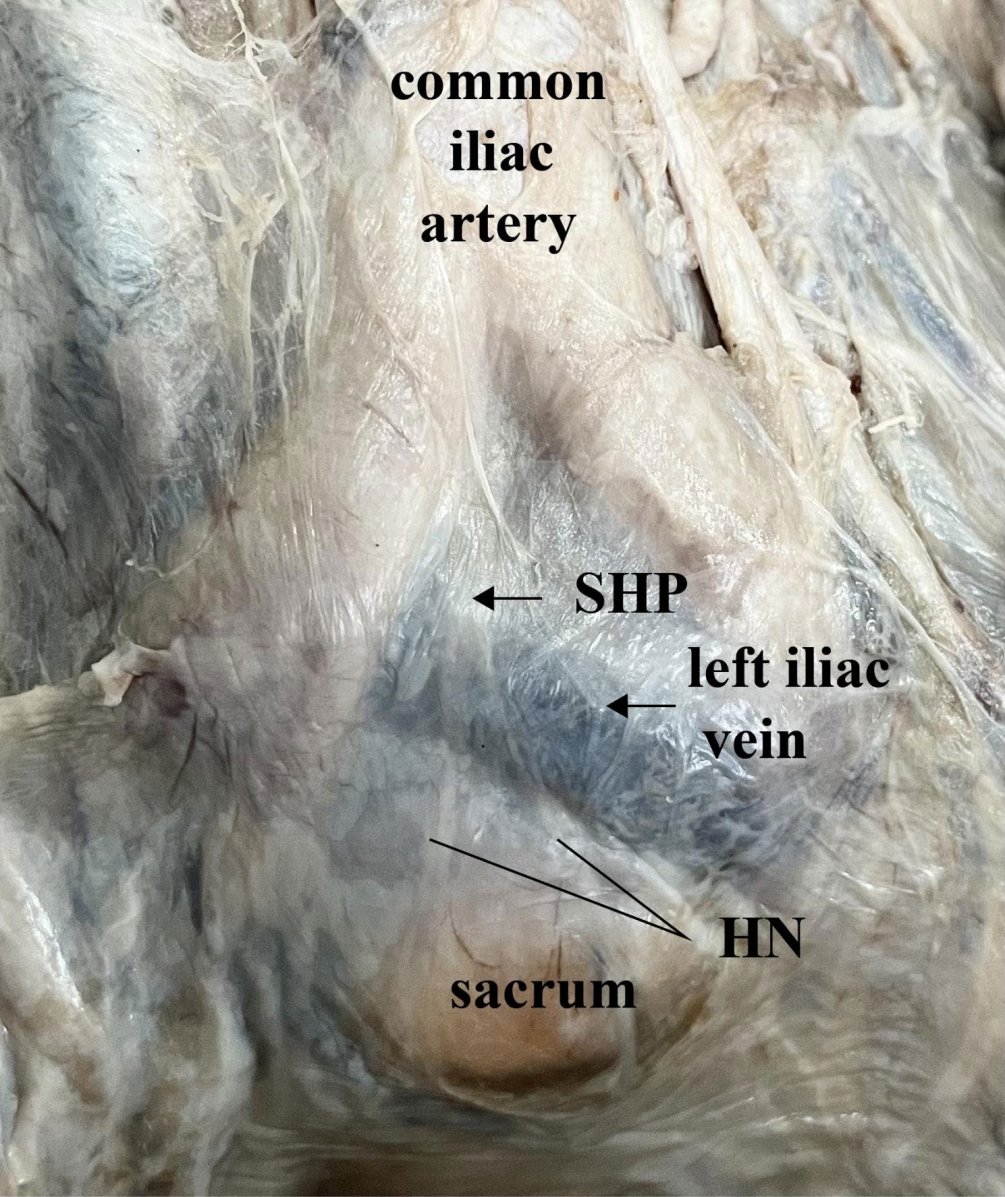
SHP on well perfused specimens

The SHP is the direct continuation of the mesenteric plexus, bright white bundles of nerve fibers that is clearly visible on well perfused specimens (Fig. 1).

**Fig. 2:**
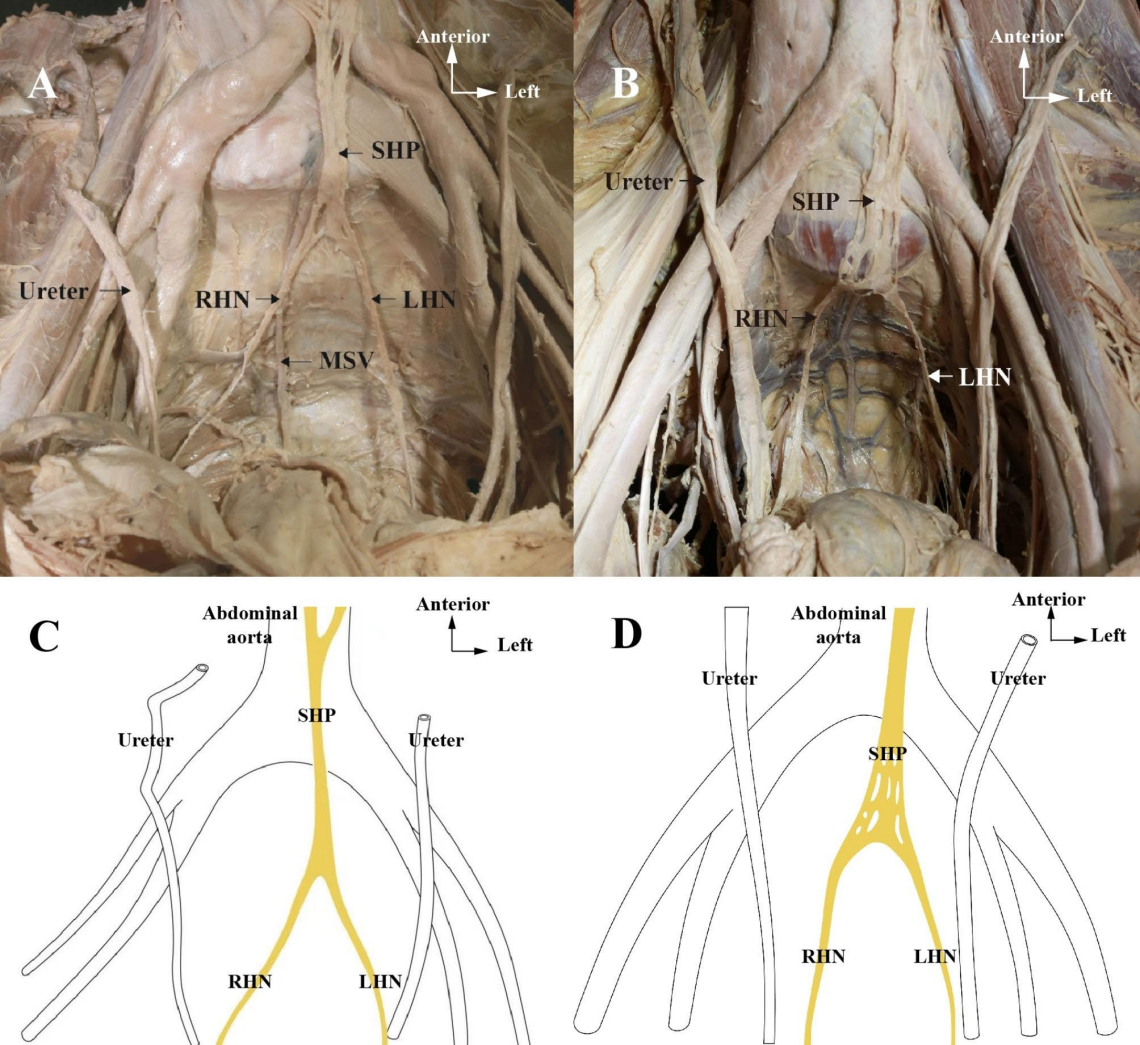
**A,B,C,D**: Morphologic variations including reticular or bundle of superior hypogastric nerves and bilateral hypogastric nerves. SHP = Superior hypogastric plexus; LHN = Left hypogastric nerve; RHN = Right hypogastric nerve; MSV = Medical sacral vein

Several morphologic variations were observed including reticular or bundle. Relationship between SHP to aortic bifurcation and abdominal midline were documented, and the distances were measured. In 60% specimens, the SHP were located left to the midline. Roughly 20% of the remaining samples had the SHP situated either in the midline or on the right-hand side of it. The ratio of the SHP located to the left and right of the midline was 3:1. The origin of bilateral HNs of all specimens were inferiorly to upper margin of promontory about 1.6 ± 0.1 cm and parallel to the ureter in front of the sacrum (Fig. 2).

### Inferior hypogastric plexus (IHP)/ pelvic plexus

The IHP was formed by contributions from the SHP, sacral splanchnic nerves, and PSNs along with the afferent fibers from pelvic viscera.

**Fig. 3:**
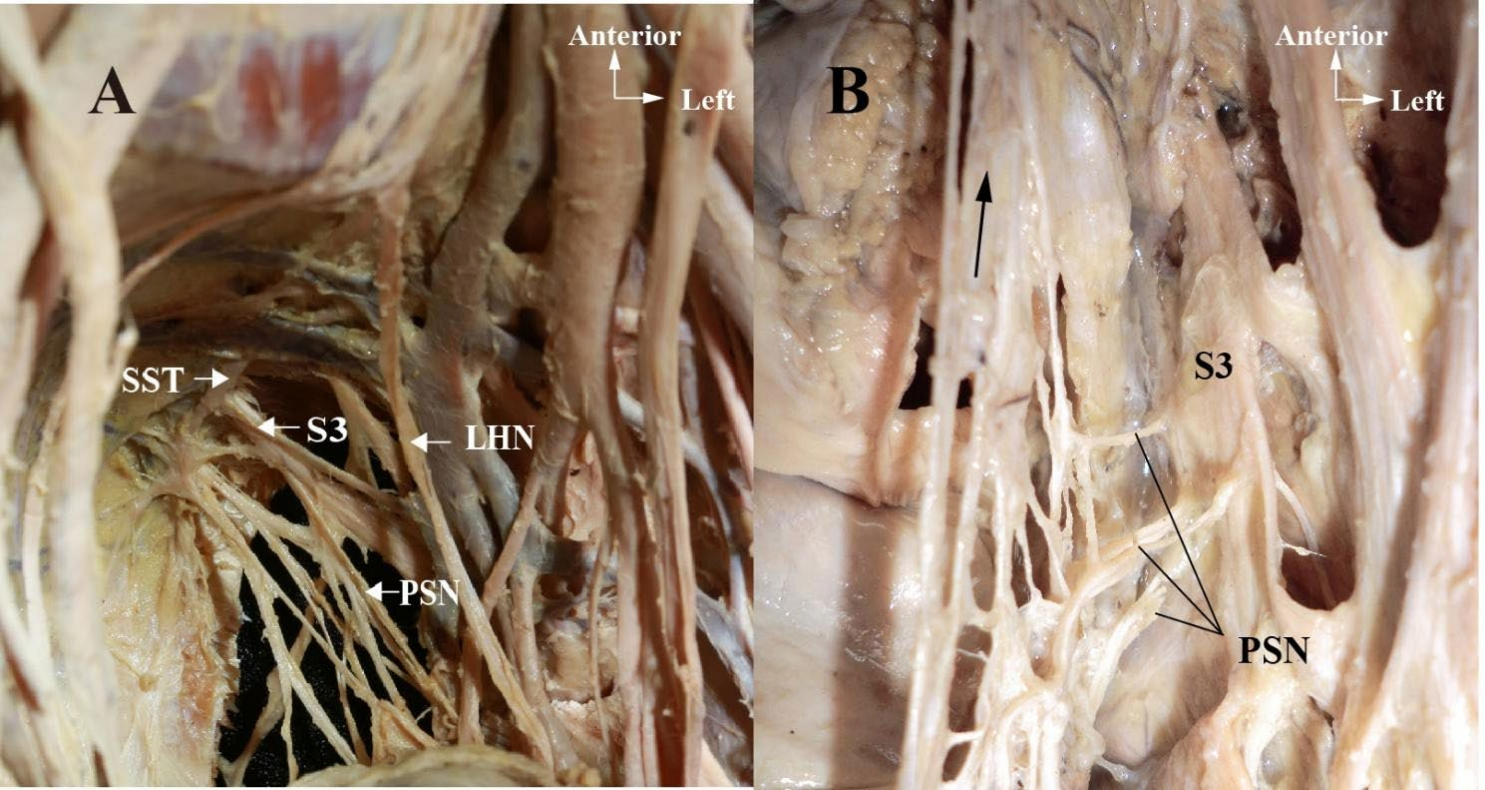
**A&B**: The course and contribution of the sacral splanchnic nerves and pelvic splanchnic nerves in the left hemipelvis. **A:** SST = Sacral sympathetic trunk; S3 = The third sacral nerve; LHN = Left hypogastric nerve; PSN = Pelvic splanchnic nerve; **B:** Arrow: Continuation of the hypogastric nerve

PSNs from S2, S3 and S4 were observed combing with the HN within the lateral rectal ligament. Particularly, the PSN from S3 was most identifiable, due to its size and stabilized location. The sacral sympathetic trunk can be identified anteriorly or medially to the anterior sacral foramen (Fig. 3).

**Fig. 4:**
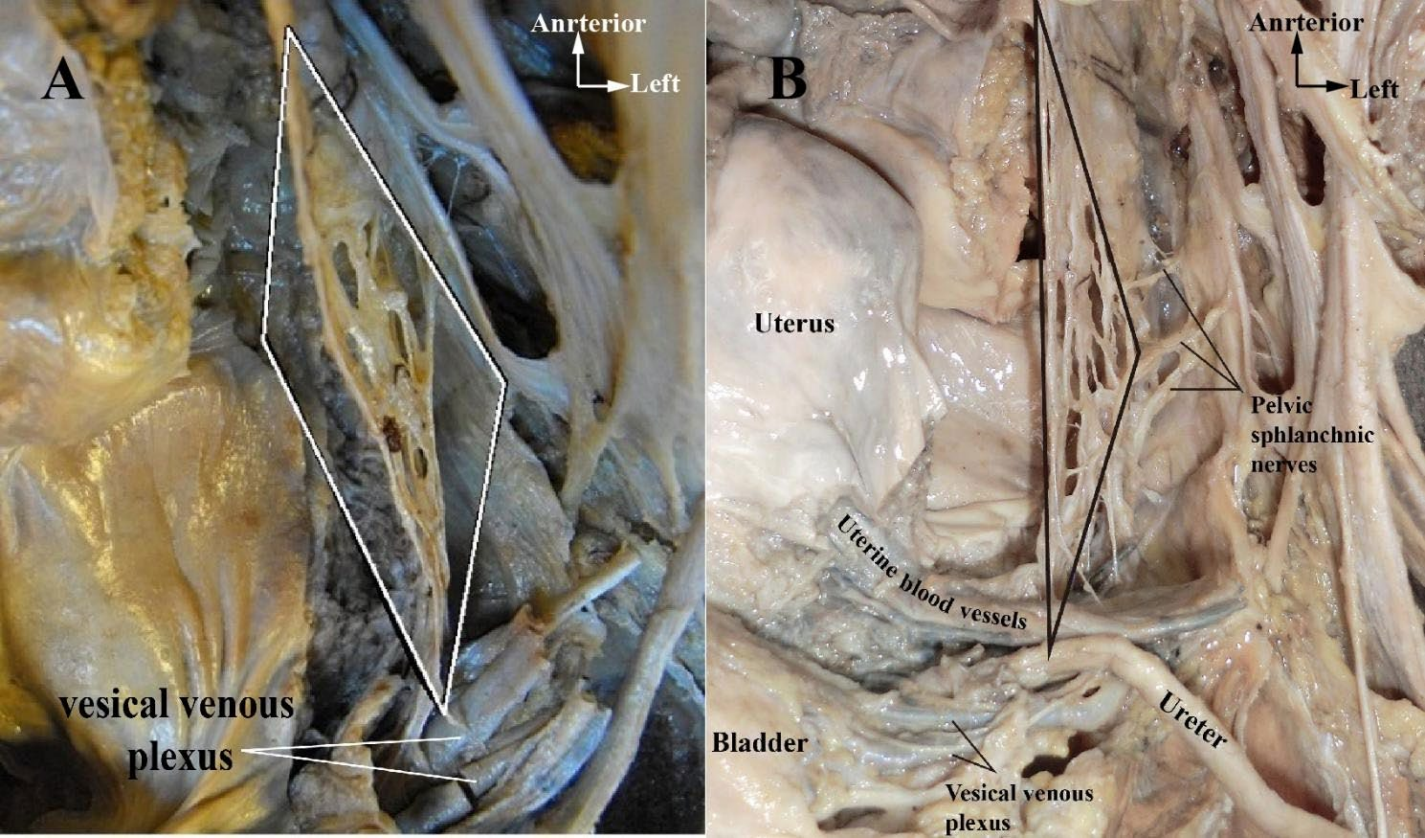
**A&B**: Morphology and boundaries of pelvic plexus in the left hemipelvis. **A:** The quadrilateral shows the morphology of pelvic plexus; **B:** The triangle shows the morphology of pelvic plexus

We identified the boundaries of pelvic plexus as following: the upper margin is formed by the PSN from S3, posterior margin by inferior rectal artery, and anteriorly by vesical venous plexus (Fig. 4).

**Fig. 5:**
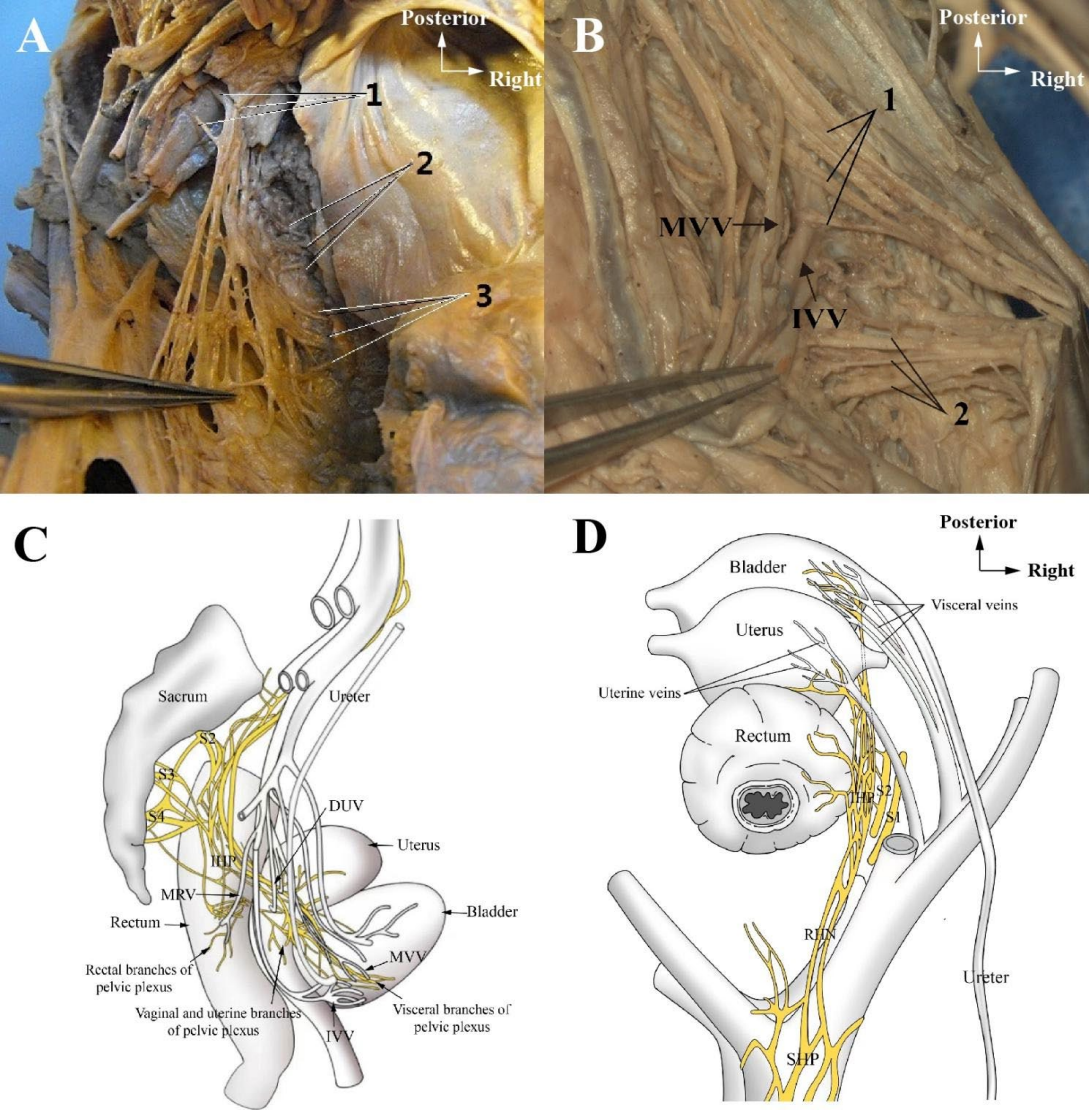
Branches of pelvic plexus(**A:** Top view of the left hemipelvis; **B:** Top view of the right hemipelvis; **C:** Side view; **D:** Top view)

The uterine branches from pelvic plexus were observed accompanying with uterine artery, while other branches were inferiorly to the artery. The PSNs were located beneath the deep uterine veins (DUV) within the cardinal ligament. The upper margin of pelvic plexus was observed directly approach to urinary bladder within the vesico-vaginal ligament as a single trunk accompanying with ureter, between the middle and inferior vesical veins, while inferior portion of bladder only receive several tiny nerves from para-uterine nervous plexus at uterine cervix level. Thus, the former may be the main innervation of bladder (Fig. 5).

A&B: 1: visceral branches of pelvic plexus; 2: Vaginal and uterine branches of pelvic plexus; 3: Rectal branches of pelvic plexus; MVV = Medical visceral vein; IVV = Inferior visceral vein.

C: S2 = The second sacral nerve; S3 = The third sacral nerve; S4 = The forth sacral nerve; IHP = Inferior hypogastric nerve; MRV = Middle rectal vein; DUV = Deep uterine vein.

D: SHP = Superior hypogastric nerve; RHN = Right hypogastric nerve; S1 = The first sacral nerve.

## Discussion

The surgical concept of preserving the pelvic autonomic nerves during radical cervical cancer surgery was first proposed by Japanese scholar Okabayashi in 1921 [13]. The procedure of NSRH was improved by anatomists and surgeons over the years [14–16]. Surgeons should be aware of the composition and course of the pelvic autonomic nerves as well as the relationship between nerves and major anatomical landmarks to achieve the radicality and safety of radical hysterectomy [17].

Great morphological differences of SHP existed between individuals. Fenestrated and cord-like shape morphology of SHP were observed, just like what Ripperda found in their study [18]. While Kutlu observed three patterns of SHP, including mesh, single and fiber, among which the mesh type is over 50% [19]. Correia et al. proposed an original morphological classification with six types, based upon the anatomical arrangement of the nervous fibers [20]. According to our observations the fibers of the SHP were located left to the midline in three-fifths of the specimens, the ratio of SHP located to the left and right of the midline was 3:1. Thus, it is suggested to carrying out an incision of the posterior peritoneal plane being along the right side of the midline and abdominal aorta in order to avoid undesirable consequences. These findings were supported by other investigators [16, 21]. It is also a matter of debate that whether SHP can be also identified as presacral nerve. The SHP comprised fibers both from the lumbar sympathetic and from thoracic sympathetic through the celiac plexus, besides, the SHP contained the terminal branches of the major and minor splanchnic nerves [22]. Thus, it’s not accurate to characterize the SHP as a single presacral nerve. As a direct continuation of SHP, the HN follows a predictable course and can be identified, dissected, and isolated during pelvic surgery, making it an important landmark to trace the visceral branches of the IHP down the HN and preserve the pelvic autonomic innervation [23].

The IHP was a mixture of nerve fibers that running in front of the sacrum and on either side of the rectum, most of it were located below the uterine vessels and was an irregular flat neural network [24, 25]. We believed that it’s not necessary to describe the exact shape of IHP, such as triangular anterior sheet or quadrilateral posterior sheet. Because its shape may change due to anatomical manipulation and artificial traction, as well as changes in the location of organs and connective tissue. The point is paying attention to the adjacent and relative positions of its branches and blood vessels and fascia [26]. Studies have shown that the uterine blood vessels and vesical veins can be used as landmarks to locate the pelvic plexus. The uterine veins can be identified in the broad ligament within the mesometrium, usually accompanied by the ascending branch of the uterine artery. The superficial uterine veins were not visible in every specimen, but the DUV always came from the uterine plexus and passed below the ureter [27]. Thus, it’s easy to identify the PSN below the DUV [28]. We suggested that the processing of the blood vessels within the cardinal ligament should be approached cautiously, in order to avoid damaging and fully expose the PSNs. The middle rectal artery (MRA) is also a key anatomical structure for surgical procedures of NSRH, although the location of the MRA was not as fixed as the location of the DUV [29], and it’s more difficult to not to damage the nerves during separating and dissecting this vessel because the MRA extends beneath and across the IHP [30], some scholars have suggested that the middle rectal artery can help to protect the IHP. Possover et al. identified the MRA as a landmark to separate the vascular from the neural part in radical hysterectomy Rutledge type III [31], the study by Centini et al. pointed out that the MRA is a landmark to identify the IHP after dissection of the right medial pararectal fossa in the surgery for endometriosis [32].

It is also important, in terms of surgical nerve preservation, to know not only the location of the pelvic plexus itself, but also the placement of the branches from the pelvic plexus to the various organs, especially the bladder [33]. The vesical veins originated from the cervix, ran beneath the uterine artery and ureter, and eventually joined the DUV [34]. Careful dissection and preservation of bladder branches is the most difficult and critical step in NSRH. During NSRH surgery, injuries of bladder branches often happened when dissecting the vesico-vaginal ligament [35]. After meticulous separation of the connective tissues in the anterior leaf of the vesico-vaginal ligament, the middle and inferior vesical veins can be appreciated in the cranial portion of the posterior leaf of the vesico-vaginal ligament [36]. The bladder branches of can be visualized after the section of middle and inferior vesical veins [37].

## Conclusions

In this study, we clarified morphological features and composition pattern of pelvic plexus as well as its related structures. We suggested the deep uterine veins and the vesical veins as land markers for clinical sugery.24 female cadaveric hemipelvis were included, consequently we could not capture the full ranges of variations. Besides, more clinical cases are needed to verify the proposed land markers for surgical procedures.

## Data Availability

All data generated or analysed during this study are included in this published article.

## List of abbreviations

NSRH: Nerve-preserving radical hysterectomy
PSNs: Pelvic splanchnic nerves
SHP: Superior hypogastric plexus
HN: Hypogastric nerve
LHN: Left hypogastric nerve
RHN: Right hypogastric nerve
IHP: Inferior hypogastric plexus
S1: The first sacral nerve
S2: The second sacral nerve
S3: The third sacral nerve
S4: The fourth sacral nerve
DUV: Deep uterine vein
MRA: Middle Rectal Artery
MSV: Medical sacral vein
MRV: Middle rectal vein
SST: Sacral sympathetic trunk
MVV: Medical visceral vein
IVV: Inferior visceral vein
